# Developmental events and cellular changes occurred during esophageal development of quail embryos

**DOI:** 10.1038/s41598-021-86503-9

**Published:** 2021-03-31

**Authors:** Soha A. Soliman, Fatma A. Madkour

**Affiliations:** 1 Department of Histology, Faculty of Veterinary Medicine, South Valley University, Qena, 83523 Egypt; 2 Department of Anatomy and Embryology of Veterinary Medicine, South Valley University, Qena, Egypt

**Keywords:** Cell biology, Developmental biology

## Abstract

The current study focused on the histogenesis of the esophagus in quail embryos. Formation of the gut tube occurred on the 4th day of incubation. Development of the muscular layers occurred in a sequential manner; the inner circular layer on the 7th day, the outer longitudinal layer on the 8th day and the muscularis mucosae on the 9th day. Glandular development began on the 13th day of incubation. The epithelium was pseudostratified columnar that consisted of mucous cells, dendritic cells, and keratinocyte precursors. Epithelial stratification occurred on the 15th day of incubation. We used Mallory trichrome, Weigert-Van Gieson, and Gomori silver stains to visualize fibrous components. Scanned samples showed formation of endoderm and mesoderm on the 5th day of incubation. A layer of myoblasts developed on the 8th day of incubation. Formation of mucosal folds, which contained glandular openings, occurred on the 14th to 17th days of incubation. On the 5th to 8th days of incubation, CD34 and vascular endothelial growth factor (VEGF) positive-mesodermal cells, and telocytes (TCs) were detected. On the 15th day of incubation, CD34 and VEGF positive-telocytes, and fibroblasts, were identified. The current study described the correlations between functional morphology and evolutionary biology.

## Introduction

The esophagus is a muscular tube that connects the pharynx to the stomach. The major differences between the avian and the mammalian esophagus are the presence of glandular lamina propria and absence of voluntary muscular movement in birds^1–3^. The avian esophagus consists of the mucosal, submucosal, and muscular layers, and adventitia. Differences in histological features of the esophagus among birds have been identified^3,4^.

The quail is considered as an ideal animal model for use in developmental biology^5^. They are easily bred in the laboratory, have a short incubation period, and rapidly reach maturation^6^. The genetic profile of the Japanese quail is almost similar to the chicken^7^. Quails have also been recently introduced as an effective laboratory bird to be used for the production of transgenic avian^8^.

Evolutionary developmental biology (EvoDevo) is defined as the *acquired* *new morphological* characters that allows the assumption of a new function responding to the environmental conditions “*adaptive zone*”^9^. Evo‐devo aims to realize the origin and evolution of embryonic development including the mechanism of development and developmental modulation that driven the *morphological* novelty, the adaptive response of development in life-history evolution, the effect of ecological changes on development to permit evolutionary modification and the developmental and phylogenetic *regulation*s of homoplasy and homology^10^. In the current study, we focused on the morphological novelties of quail esophagus including absence of ciliated epithelium and striated muscles in quail esophagus, alternative development of surface mucous cells/esophageal glands, short glands and shallow submucosa. We also the compared the morphological features of quail esophagus to other avian, mammalian, fish, reptilian species and discussed the possible role in adaptation to their environments.

Determining the histogenesis of different organs is necessary to gain insight and basic knowledge about the accurate timing of the histological events that occur during embryonic development. Quails may be used to study esophageal motility disorders such as achalasia and esophageal atresia. The current study focused on the histological development of the esophagus in quail embryos.

## Material and methods

### Sample collection and preparation of paraffin sections

Fertile quail (Coturnix Coturnix japonica) eggs were obtained from a farm belonging to the Department of Histology, Faculty of Veterinary Medicine, South Valley University, Qena, Egypt. Incubation of eggs was performed according to protocol^11^. Eggs were incubated at 37.5 °C with a relative humidity 65%. Quail embryos were collected from the 4th to the 17th days (final day) of incubation. Embryonic stages were determined upon the onset of incubation.

Sample collection and fixation for preparation of paraffin embedding were performed according to instructions^11^. The eggs were opened from the wide end by using a hand scalpel, then the embryos were carefully pulled from the egg shells. We used 2 petri dishes; one during egg opening and the other for placing the embryos, which were washed with distilled water then saline solution (0.9% NaCl). Embryos at days 3–13 of incubation were kept at minus 20 °C for 4 h prior to collecting, while days 15–17 were sacrificed and their skin incised from the recti of beak to the thoracic inlet, allowing access to the body cavity. Sample collection was done following the guidelines of the Institutional Ethical Committee of Veterinary Medicine, South Valley University, Egypt, and following the Egyptian Animals' laws. Bouin’s fixative (Table 1)^12^ was used for embryo fixation. Five embryos were collected for each incubation age and immersed in Bouin’s solution for 24 h. Embryos at days 15 and 17 of incubation were decalcified using 10% ethylenediaminetetraacetic acid. The protocol of the study was approved by the Egyptian Research Ethics Committee. Whole embryos were processed for preparation of paraffin sections, and was performed according to instructions^13^. Samples were dehydrated using ascending grades of ethanol (Table 1), cleared using methyl benzoate (Table 1), and embedded in paraffin wax (Table 1). Paraffin sections were sliced using Richert Leica RM 2125 Microtome, Germany.

**Table1:** The processing time of the samples in paraffin embedding techniques.

**1-Fixation**	
1-F A-NBF	24 h
B_Bouin’s solution	1/2 h
**2-Dehydration**	
Alcohol70%I	2 h
Alcohol 70%II	2d
Alchol70%III	2 h
Alchol80%	1 h
Alchol90%	1/2 h
Alchol100%	1/2 h
**3-Clearing with methylebenzot**	
MBI	1 h
MB II	12 h
MBIII	12 h
**4-Embedding in paraffin**	
P I	1/2 h
P II	1/2 h
PIII	1 h

*NBF* (neutral buffer formalin), *h* hours, *d* days, *MB I* methyl bonzoate1, *MB II* methyl benzoate II, *PI* paraffin I, *P II* paraffin II, *P III* paraffin III.

### Histochemical staining

For histological examination, paraffin sections were stained by hematoxylin and eosin (H&E)^14^. Mallory trichrome stain was used to visualize collagen fibers and the muscles, and was performed according to instructions^15^. Bielschowsky’s silver stain was used to visualize myofilaments, and was performed following instructions as indicated^16^. Gomori silver stain was used for visualization of reticular fibers, and was performed according to instructions^17^. Weigert-Van Gieson stain was used according to instructions^12^.

The stained sections were examined using a Leitz Dialux 20 microscope. Photos were taken using a Canon digital camera (Canon Powershot A95).

### Immunohistochemistry (IHC) staining using CD34

Samples used for IHC investigations were fixed in NBF (neutral buffered formalin) (Table 1). Detection of antigen localization was performed using a combination of the avidin–biotin complex technique^18^ and the reagent of the Ultra Vision Detection System (Anti-Polyvalent, HRP/DAB manufactured by Thermo Fisher Scientific TP-015HD). The procedures were performed according to the manufacturer’s instructions^19–21^. Paraffin sections measuring 5 µm were dewaxed by xylene, hydrated with ascending grades of alcohol, and washed for 5 min twice with PBS (phosphate buffered saline) at pH 7.4 (Table 1). The sections were treated with 3% hydrogen peroxide in methanol at room temperature for 20 min to block endogenous peroxidase activity. The sections were rinsed for 10 min with running tap water. Improving antigen retrieval requires use of a 10 mm sodium citrate buffer (pH 6.0) (Table 2) in a water bath for 20 min at 95–98 °C. This was followed by slide cooling for 20 min at room temperature, and subsequently washed for 5 min twice with PBS at pH 7.4. Ultra V block was used for 5 min at room temperature to prevent nonspecific background staining. To avoid artifacts, use of ultra V block should not exceed 10 min. The primary antibody (Table 3) was applied on the sections overnight at 4 °C. The sections were then washed for 5 min thrice with PBS at pH 7.4. The biotinylated secondary antibody (Goat Anti-Polyvalent, Anti-Mouse IgG + Anti -Rabbit IgG; Thermo Fisher Scientific, UK; Lab Vision Corporation; Table 2) was applied on the sections for 10 min at room temperature. The sections were then washed for 5 min twice with PBS at pH 7.4 and incubated for 10 min at room temperature using streptavidin-peroxidase complex (Thermo Fisher Scientific, UK; Lab Vision Corporation, USA). Visualization of the bound antibodies was carried out by incubating the section in a humid chamber using a mixture of 1 drop of 3,3′-diaminobenzidine (DAB) and chromogen (Table 4) to 2 mL of DAB plus substrate for 5 min at room temperature. The sections were counterstained with Harris hematoxylin for 30 s. The sections were dehydrated using ethanol and isopropanol I and II, cleared in xylene, and covered by DPX. CD34 immunohistochemical staining was examined using the Leitz Dialux 20 microscope provided with the Canon (Power shot A95) digital camera.

**Table 2:** Components of the fixative.

Fixative	Components	Amount
Karnovsky fixative	Paraformaldehyde, 25% freshly prepared	10 mL
	Glutaraldehyde 50%	10 mL
	Na-Phosphate buffer (0.1 M, pH 7.4)	50 mL
	Distilled water	30 mL
N a-phosphate buffer (0.1 M, pH 7.4)	Solution A	
	Na_2_HPO_4_ 2H_2_O	17.02 gm
	Distilled water	600 mL
	Solution B	
	NaH2PO4 H2	6 gm
	Distilled water	200 mL
	Using solution	
	Solution A	580 mL
	Solution B	219 mL
Citrate-buffer (pH 6.0)	Solution A	
	Citrate C_6_H_8_O_7_ H_2_O	21 g
	Distilled water	1 L
	Solution B	
	Sodium citrate Na_3_C_6_H_5_O_7_ 2H_2_O	29.41 g
	Distilled water	1 L
	Using solution	
	Solution A	9 mL
	Solution B	41 mL
	Distilled water	Add 500 mL

**Table 3:** Identity, sources, and working dilution of antibodies used in immunohistochemical studies.

Target	Primary antibody supplier	Origin (catalog no)	Dilution	Incubation	Antigen retrieval	Secondary antibody-incubation time
CD34	MOUSE ANTI CHICKEN CD34 (Bio rad)	MOUSE ANTI CHICKEN CD34 Monoclonal Antibody (Clone: AV138) (Cat.no MBS224490)	1:100	Over night	Boiling in citrate buffer (pH 6.0), 20 min Goat	Goat anti-Mouse IgG (H+L) Secondary Antibody Catalog # 31,569 Dilution ; 1:100 One hour at room temperature
VEGF	Rabbit anti -VEGF (Invitrogen by Thermo Fisher Scientific Waltham, MA, USA))	Rabbit VEGF Polyclonal Antibody (clone: RB-222-P0) (Cat.no PA1-21,796)	1:100	Over night	Boiling in citrate buffer (pH 6.0), 20 min	Goat anti-rabbit secondary antibody (cat. no. K4003, EN Vision+ TM System Horseradish Peroxidase Labelled Polymer; DAKO) Ready to use 30 min at room temperature

Antibodies used that showed reactivity in Avian species.

**Table 4:** Reagent of ultra vision detection system (anti-polyvalent, HRP/DAP) for CD34.

Tp-015-HD	Component
TA-015-HP	Hydrogen peroxide block
TP-015-UB	Ultra V block
TP-015-BN	Biotinylated goat anti-polyvalent
TS-015-HSX	Streptavidin peroxidase
TP-015-HCX	DAB plus substrate
TA-015-HP	DAB plus chromogen

### Immunohistochemical procedures of vascular endothelial growth factor (VEGF)

Samples used for VEGF immunostaining were fixed in NBF (neutral buffer formalin) (Table 1). The two-step immunohistochemical staining procedures utilized the DAKO EN Vision TM and System, HRP peroxidase^22^. The staining was performed according to instructions^23^. Paraffin sections of 5 µm were dewaxed, rehydrated, and rinsed for 5 min twice with PBS at pH 7.4. Blocking of the endogenous peroxidase activity was achieved by using drops of 3% hydrogen peroxide in methanol for 20 min at room temperature, then thoroughly washed with running tap water for 10 min. Antigen retrieval was performed using 10 mm sodium citrate buffer (pH 6.0) (Table 2). The buffer was heated to 95 °C–98 °C in a water bath for 20 min, followed by cooling at room temperature for 20 min. Sections were washed for 5 min twice with PBS at pH 7.4. Blocking nonspecific background staining was performed using drops of blocking serum (DAKO) to cover the sections for 5 min at room temperature. The sections were then incubated with the primary antibody. The antibodies that were applied showed immunoreactivity in avian species^24^. Table 3 explored the identity, sources, and the working dilution of antibodies used in the immunohistochemical technique. The slides were rinsed for 5 min thrice with PBS at pH 7.4 then incubated with secondary antibody for 30 min at room temperature. The slides were again washed for 5 min twice with PBS at pH 7.4 and incubated for 5–10 min at room temperature with 3,3′-diaminobenzidine (DAB) and substrate-chromogen that produced a brown color at the antigen site. The slides were then counterstained with Harris hematoxylin for 30 s. The sections were dehydrated with ethanol alcohol 90%, then 100% II, cleared in xylene, and covered using DPX. VEGF immunohistochemically-stained sections were examined using the Leitz Dialux20 microscope provided with the Canon (PowerShot A95) digital camera -vecontrols were performed with the same procedures except using primary antibodies.

### Preparation of samples for embedding in resin

Resin embedding technique was performed according to^25^ using Karnovsky’s fixed samples. Five samples were used from incubation days 5, 8, and 15. Each esophagus, measuring 2–3 mm in length, was carefully excised after sacrificing the quail. Karnovsky fixative^26^ was prepared from a mixture of 10 mL of 25% paraformaldehyde, 10 mL of 50% glutaraldehyde, 50 mL phosphate buffer, and 30 mL distilled water. Karnovsky fixative was applied overnight at 4 °C. The samples were post-fixated by osmium tetroxide, dehydrated using ascending grades of alcohol, inoculated in a mixture of alcohol/resin and pure resin, resin embedding and crystallization was performed in oven at 60 °C degrees. Semi-thin sections (1 μm) were created using an ultramicrotome (Ultracut E, Reichert-Leica, Germany) and stained with toluidine blue^27,28^, methylene blue^29,30^, and periodic acid-Schiff (PAS)^31^. Staining of semi-thin sections required dissolving of resin using saturated alcoholic solution of sodium hydroxide. The stained sections were examined using a Leitz Dialux 20 microscope and a Canon digital camera (Canon PowerShot A95).

### Transmission electron microscopy

Ultra-thin Sects. (60 nm) of the esophagus at incubation days 8, 13, and 15 were taken by using a Reichert ultramicrotome. The sections were stained with uranyl acetate for 15 min then lead citrate for another 15 min. The stained grids were examined by using a JEOL100CX II transmission electron microscope at the central laboratory of South Valley University, Egypt.

### Scanning electron microscopy

The samples were washed several times with normal saline and then fixed in 4% glutaraldehyde solution for 24 h at pH 7.3. They were then washed in sodium phosphate buffer at pH 7.3 4 times for 15 min and fixed in 1% osmic acid and 0.1 M sodium phosphate buffer for 2 h at room temperature. Thereafter, they were washed with 0.1 M sodium phosphate buffer 4 times for 15 min. The samples were then dehydrated with 50%, 70%, and 90% alcohol for 30 min in each concentration, then 100% for 2 days followed by isoamyl acetate for another 2 days. The samples were subjected to the critical point drying method (Critical Point Drying Procedure Polaron E3000 CPD Apparatus, Germany) and coated with gold using JEOL-1100 E-ion Sputtering Device (Japan). Samples were examined using a JEOL scanning electron microscope (JSM 5500 LV) at 10 kV at the central laboratory of South Valley University, Egypt.

### Coloring of TEM and SEM images

We colored the images produced from the transmission (TEM) and scanning (SEM) electron microscopes using the Photo Filter 6.3.2 program to recognize different types of cells and to distinguish different structures. The methods used were as previously described^19,30,32–50^.

### Ethical approval

The National Ethics Committee of South Valley University and veterinary authorities in South Valley University Province, Egypt, approved the method of this study. ‘All procedures were performed in accordance with the relevant guidelines and regulations’^51^. We stated that the study was carried out in compliance with the ARRIVE guidelines in Ethics approval section.

## Results

**Figure 1:**
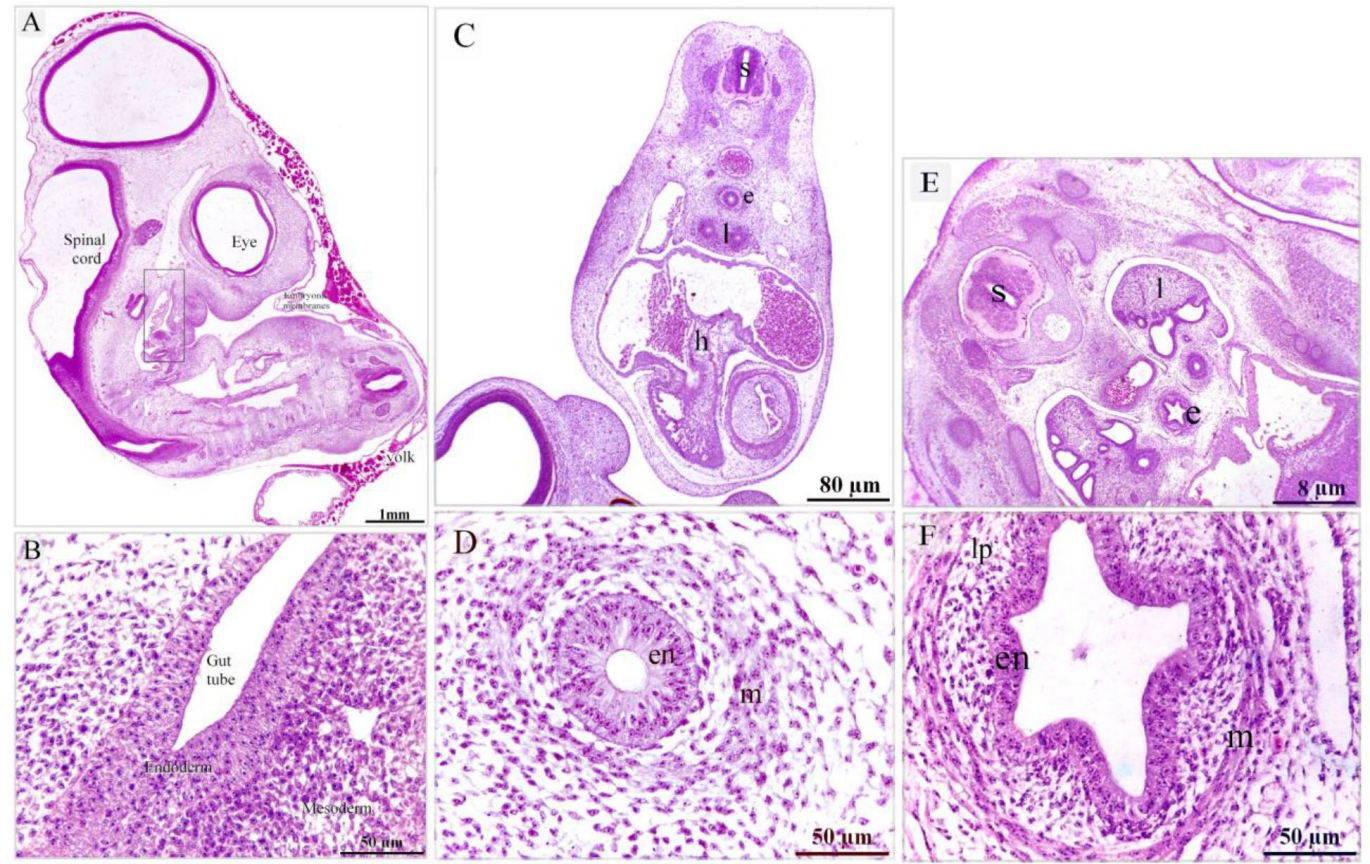
Formation of the gut tube and development of the circular muscular wall. Paraffin sections of day 4 (**A**, **B**), 5 (**C**, **D**), day 6 (**E**–**G**) of quail embryo stained with hematoxylin and Eosin (H&E). (**A**) The whole embryo developed the gut tube (the squared area). (**B**) Higher magnification of the gut tube show endoderm covered by mesoderm. (**C**) Spinal cord (s), primitive esophagus (e), lung buds (lu), heart (h). (**D**) Higher magnification of the initial part of the foregut; the primitive esophagus. Note Mesenchymal condensation (m) which represents muscular wall development, endoderm (en) of pseudostratified type of the epithelium. (**E**) Spinal cord (s), lung (l), esophagus (e). (**F**) Esophagus developed the circular muscular layer (m) of smooth muscle cells. The mucosa was folded and consisted of endodermal cells (en) of pseudostratified epithelium and lamina propria (lp).

**Figure 2:**
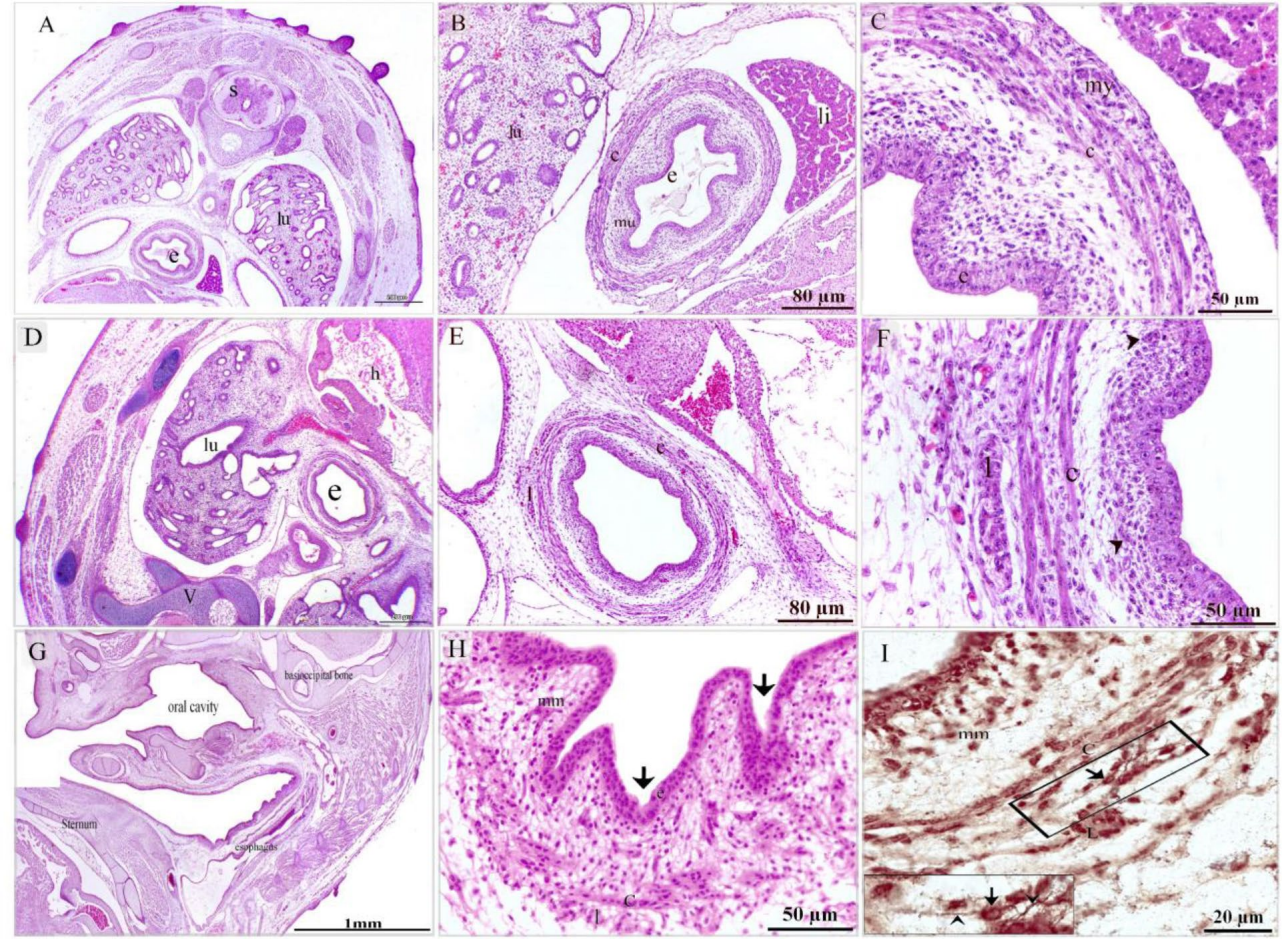
Development of the muscularis mucosae and the longitudinal layer of the muscular sheath. Cross section of day 7 (**A**–**C**), 8 (**D**–**F**), 9 (**G**–**I**) of quail embryo stained with H&E (**A**–**H**) and Bielschowsky's Silver (**I**). A: spinal cord (s), lung (lu), esophagus (e). B: esophageal wall consisted of folded mucosa (mu), muscular layer (c), Lung (l), liver (li) C: endodermal cells (en), distinctive circular layer of smooth muscle cells (c), aggregation of myoblasts (my) of the prospective longitudinal muscular layer. (**D**) Esophagus (e), heart (h), lung (lu), vertebrae (v). (**E**, **F**) The esophagus developed distinct inner circular (c) and outer longitudinal (l) muscular layers. Note aggregated mesenchymal cells (arrowheads) in the mucosal layer. (**G**) The esophagus had folded mucosa. (**H**) the esophagus exhibited distinct muscular bundles, the inner circular (c) and outer longitudinal (l) muscular layers, muscularis mucosae (mm). Mucosal folding (arrow). (**I**) The wall of the esophageal muscular bundles, the inner circular (c) and outer longitudinal (l), muscularis mucosae (mm). Note telocytes (arrow).

**Figure 3:**
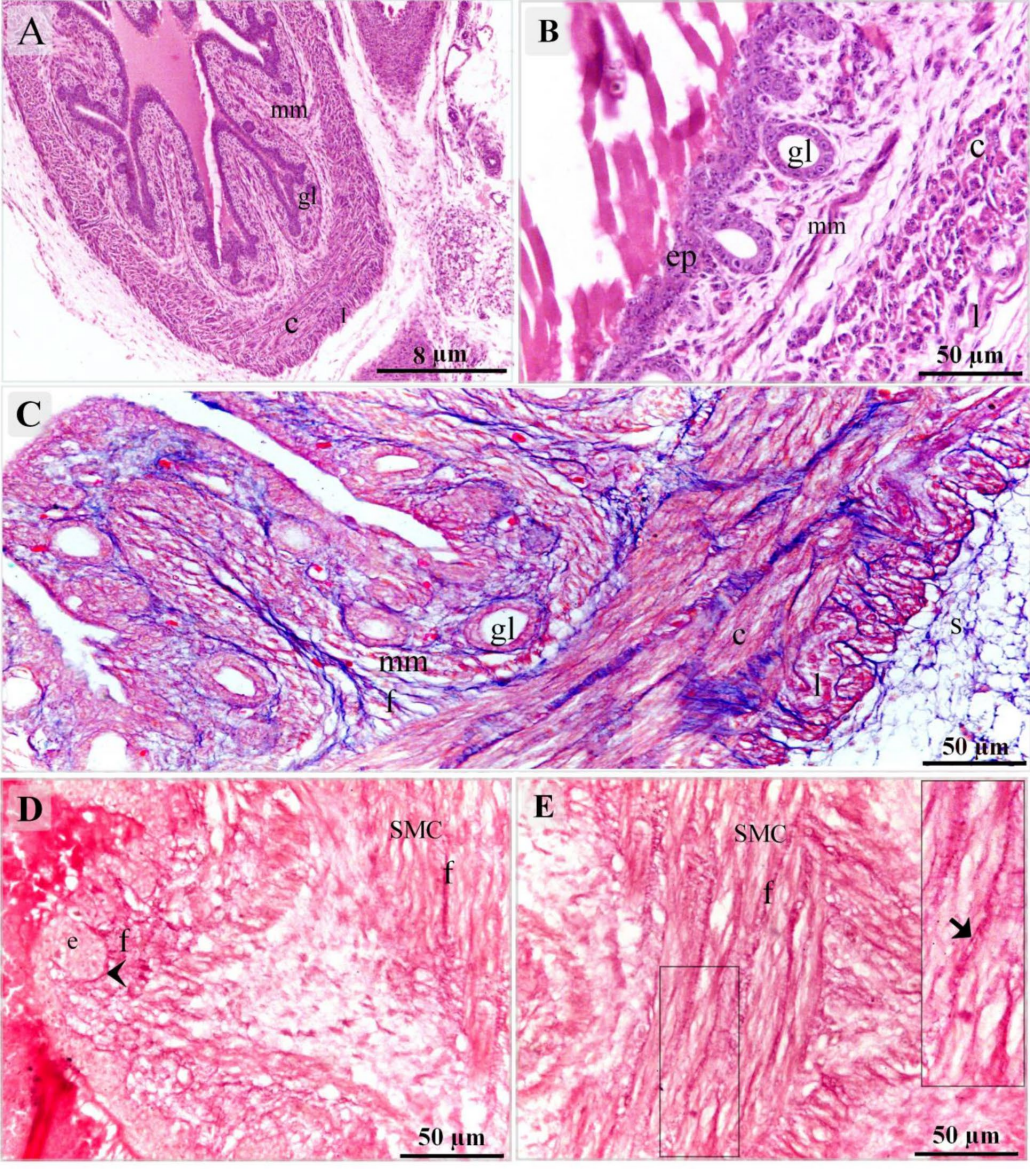
Initiation of glandular development. Cross section of day 13 of quail embryo stained with H&E (**A**, **B**), Mallory trichrome stain (**C**), Weigert-Van Gieson stain (**D**, **E**). (**A**) Invagination of the sac-like glandular units (gl), thickening of the inner circular (c) and outer longitudinal (l) muscular layers, muscularis mucosae (mm). (**B**) Esophagus lined by pseudostratified epithelium (ep), the sac-like glandular units (gl), the inner circular (c) and outer longitudinal (l) muscular layers, muscularis mucosae (mm). (**C**) The esophagus had a distinct muscular layer; The inner circular (c) and outer longitudinal (l) muscular layers. Collagen fibers (f). Note muscularis mucosae (mm), serosa (s) gland (gl). (**D**, **E**) Collagen fiber-rich (f) connective tissues were located under the epithelium (e) and between the muscle bundles (SMC). Collagen fibers located in the basal lamina (arrowhead). Note telocytes (arrow).

On the 4th day of incubation, the gut tube was formed and consisted of endoderm and covered by mesoderm (Fig. 1A,B). On the 5th day of incubation, the primitive esophagus had an endodermal layer of pseudostratified epithelium and surrounded by a condensed layer of mesenchyme, a marker for the development of a muscular wall (Fig. 1C,D). This muscular wall began to develop on the 6th day of incubation. A circular layer of SMC (smooth muscle cells) was observed (Fig. 1E,F). On the 7th day of incubation, the circular muscular layer became more distinct while myoblasts aggregated to form the primitive outer longitudinal muscular layer (Fig. 2A–C). On the 8th and 9th days of incubation, the esophagus developed distinct inner circular and outer longitudinal muscular layers (Figs. 2A–I). The muscularis mucosae recognized on the 8th day of incubation by mesenchymal condensation and identified as a thin layer of SMC that supports the mucosa on the 9th day of incubation. (Fig. 2H). On the 13th day of incubation, the esophagus developed muscualris mucosae rather than the distinctive inner circular and outer longitudinal muscular layers (Fig. 3A–D). The epithelial invaginations formed sac-like glandular units. The epithelium was pseudostratified type (Fig. 3A,B). Presence of collagen fibers in the lamina propria, between the muscle fibers, and the serosa were visualized using Mallory trichrome (Fig. 3C) and Weigert-Van Gieson stains (Fig. 3D,E).

**Figure 4:**
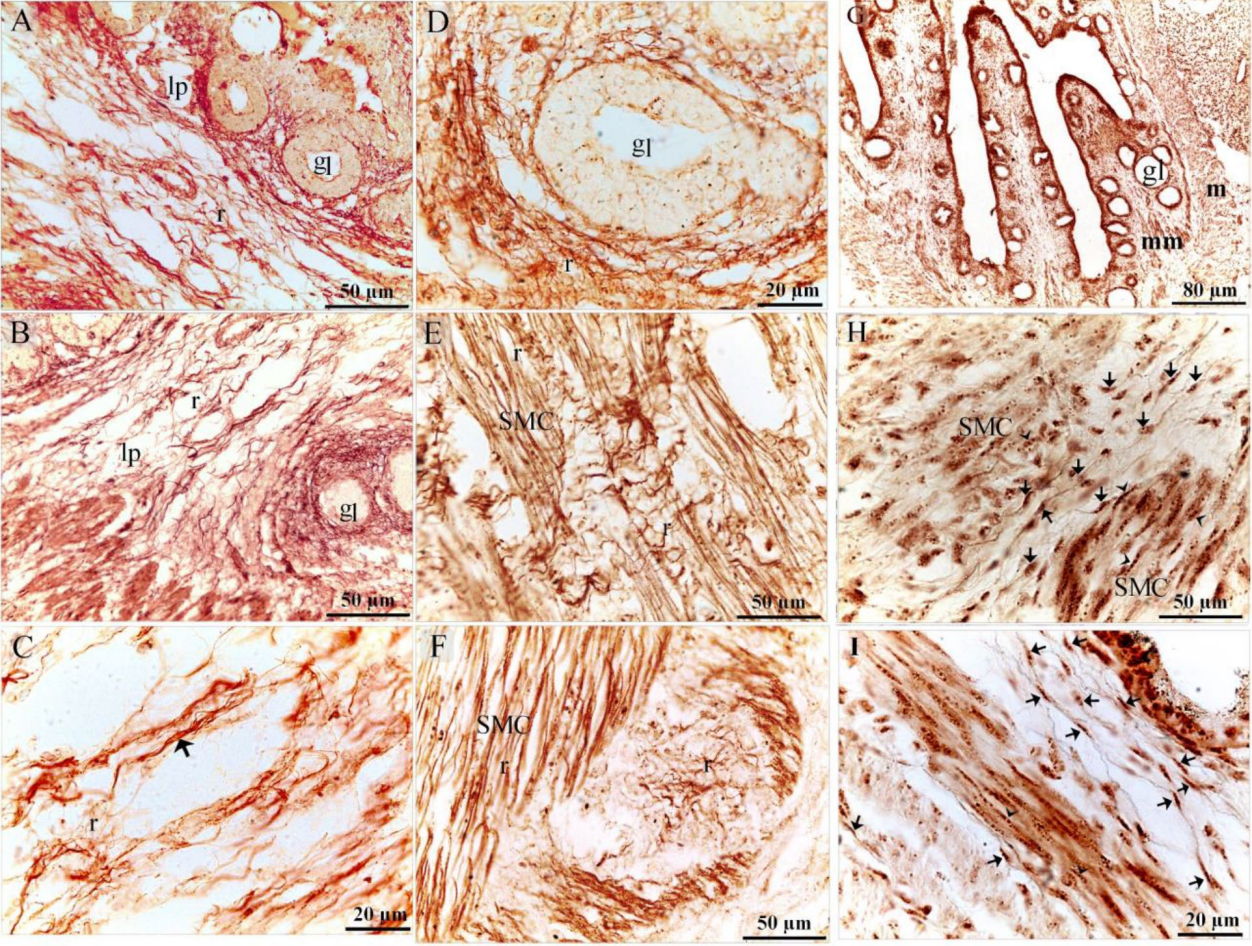
Detection of the reticular fibers using Gomori's silver stain. Cross section of day 15 of quail embryo stained with Gomori's silver stain (**A**–**F**) and Bielschowsky's silver stain (**G**–**I**). (**A**, **B**) reticular fibers (r) in the lamina propria (lp). Note gland (gl). (**C**) telocyte (arrow) located in the LP. Note reticular fibers (r). (**D**) Reticular fibers (r) surrounded the gland (gl). (**E**, **F**) Reticular fibers (r) surrounded each cell of smooth muscle (SMC) and in the myenteric or Auerbach's plexus. (**G**–**I**) Telocytes (arrows) between the muscle bundles (SMC). Note granular appearance indicating dense bodies (arrowheads).

**Figure 5:**
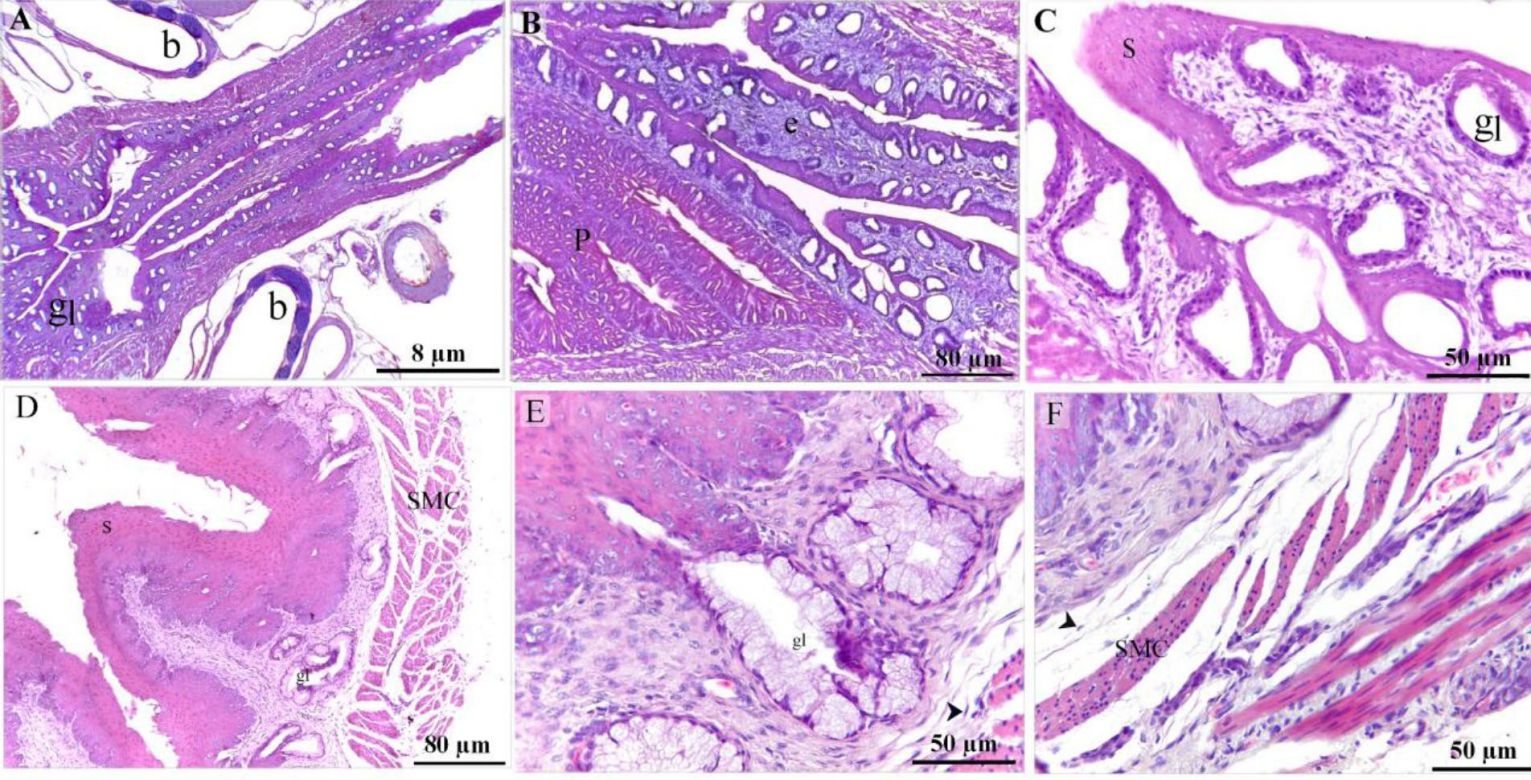
Typical form of avian esophagus. Coronal section of day 15 of quail embryo stained with H&E (**A**–**F**). A, B: The thoracic portion of the esophagus (e) developed highly folded mucosa which exhibited numerous sac-like glandular units (gl). intrapulmonary bronchi (b). Note. proventriculus (P). (**C**) The thoracic portion of the esophagus was lined by stratified squamous epithelium non-keratinized (s), inactive glands (gl). (**D**–**F**) The cervical portion developed active mucous esophageal glands (gl). Note stratified squamous epithelium non-keratinized (s), muscular layer (SMC). Telocytes (arrowheads).

On the 15th day of incubation, reticular fibers were recognized using Gomori’s silver stain in the lamina propria (Fig. 4A,B), around the gland (Fig. 4D), around smooth muscle cells and in the myenteric or Auerbach's plexus (Fig. 4E,F). TCs were identified in the LP (Fig. 4C) and between the muscle bundles (Fig. 4G–I). Bielschowsky's silver stain was used to visualize the SMC, which revealed a granular appearance indicating the presence of dense bodies (Fig. 4G–I). The thoracic portion of the esophagus developed highly folded mucosa which exhibited numerous sac-like glandular units, inactive glands (Fig. 5A,B) the epithelial lining had stratified squamous epithelium non-keratinized (Fig. 5C). The cervical portion also had stratified squamous epithelium non-keratinized developed active mucous esophageal glands (Fig. 5D–F). Telocytes were recognized in the lamina propria (Fig. 5E) and submucosa (Fig. 5F).

**Figure 6:**
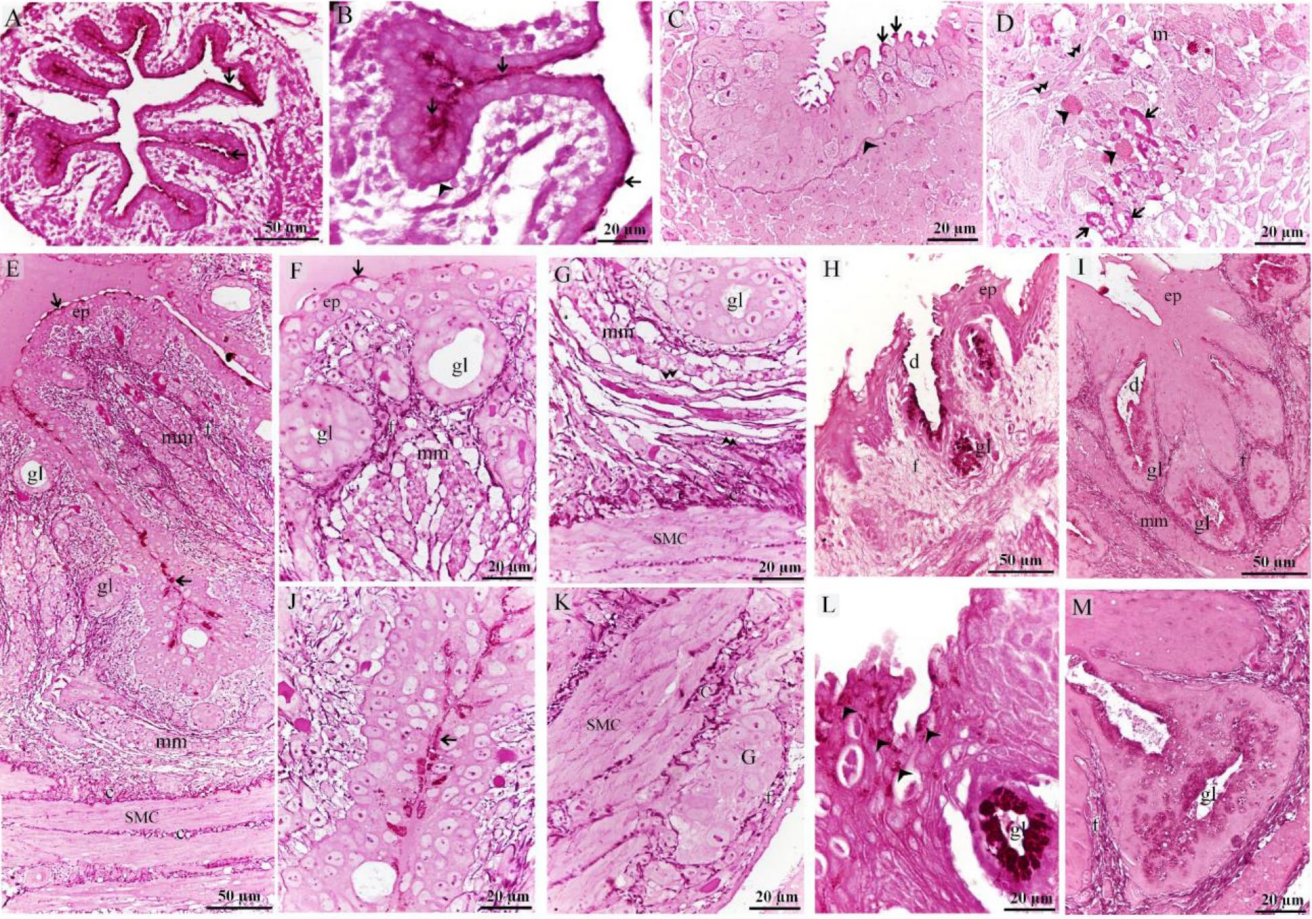
Staining affinity of the day 8, 13, 15 esophagus for PAS. Paraffin (**A**, **B**, **E**, **J**) and semi-thin (**C**, **D**, **F**–**I**, **K**–**M**) sections of day 8 (**A**–**D**), 13 (**E**–**G**, **J**, **K**) and 15 (**H**, **I**, **L**, **M**) stained with PAS. (**A**–**C**) epithelium had PAS-positive granules (arrows). basal lamina (arrowheads). (**D**) The developing muscular layer was distinguished by the myoblasts (m). note PAS-positive glycogen granules (arrowheads), PAS-positive myelin sheath (arrows). Telocytes (double arrowheads) located around the developing muscular layer. (**E**–**G**, **J**, **K**) The esophagus was lined by pseudostratified epithelium which exhibited PAS-positive reaction at the apical surface (arrows). The gland (gl) was inactive PAS-ve. Note PAS-positive Reticular fibers (f). muscularis mucosae (mm). muscular layer (SMC). parasympathetic ganglion (G), Telocytes (double arrowheads). (**H**, **I**, **L**, **M**) The esophagus was lined by stratified squamous epithelium (ep) and developed active mucus glands (gl) that exhibited PAS-positive granules. The duct (d) was lined by mucous-secreting cells (arrowheads). Note PAS-positive Reticular fibers (f). muscularis mucosae (mm).

**Figure 7:**
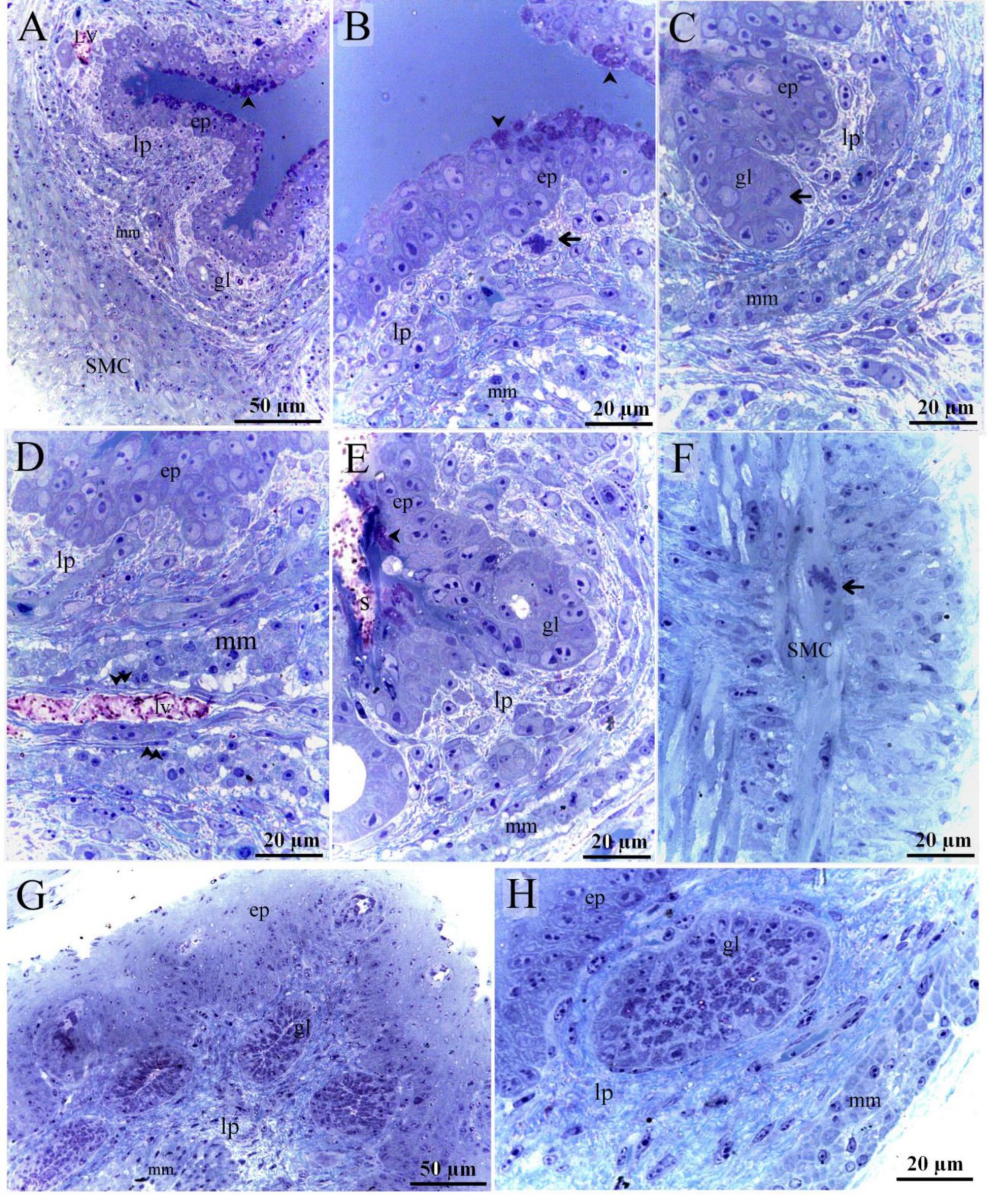
Semi-thin sections of days 13 and 15 stained by toluidine blue. Semi-thin sections of days 13 (**A**–**F**) and 15 (**G**, **H**) stained with toluidine blue. (**A**–**F**) The esophagus lined by pseudostratified epithelium (ep) which contained mucous producing cells. Note metachromatic granules (arrowheads), inactive gland (gl), lamina propria (lp), muscularis mucosae (mm), smooth muscle cells of tunica muscularis (SMC), blood vessels (bv), Telocytes (double arrowheads). Metachromatic section (s) at the epithelial surface and inside the lymph vessels (lv). Mitotic divisions (arrows). (**G**, **H**) The esophagus developed stratified squamous epithelium (ep) and active mucous glands (gl). Note lamina propria (lp), muscularis mucosae (mm).

**Figure 8:**
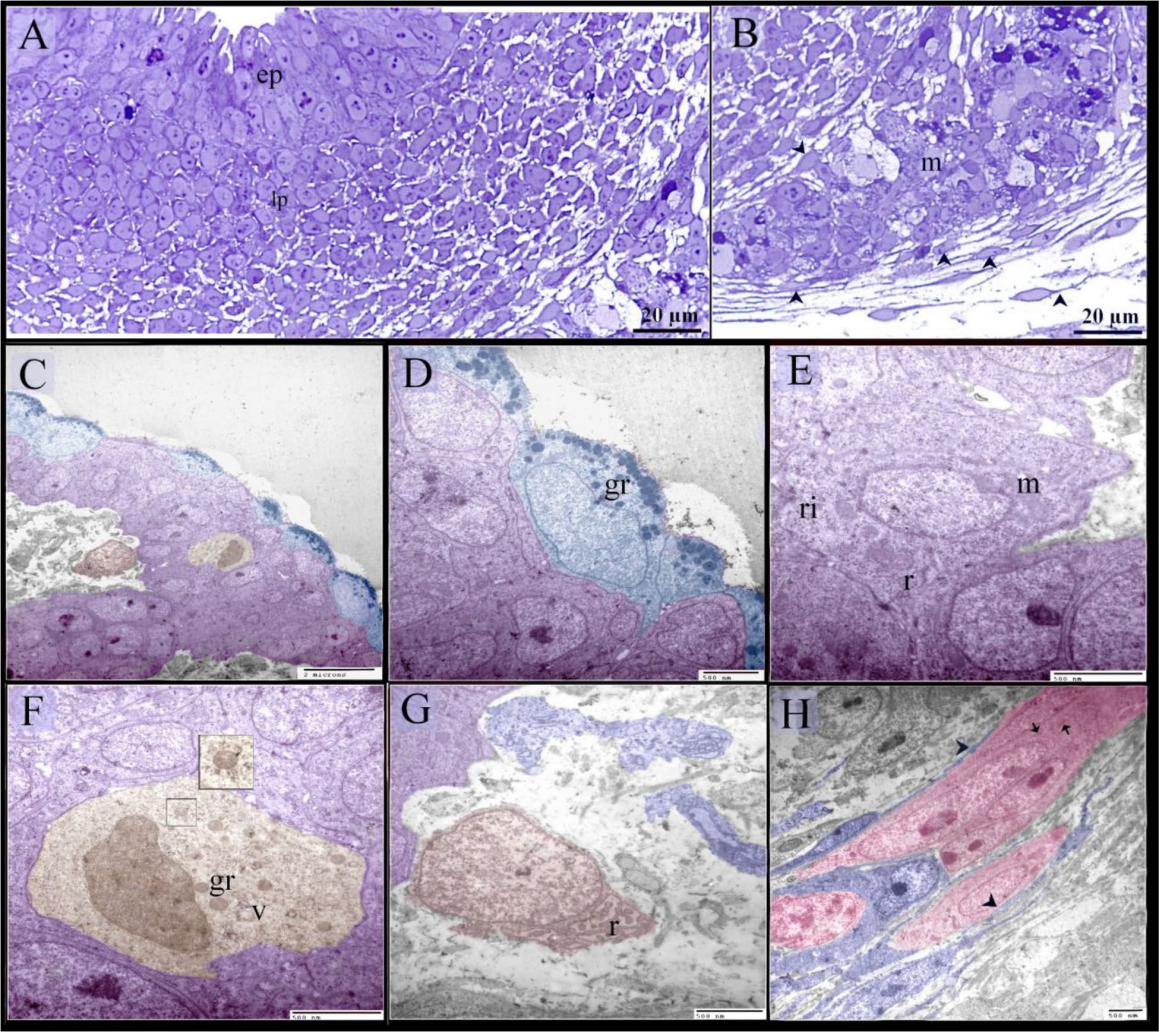
Semi-thin sections and ultrastructure at day 8. Semi-thin sections (**A**, **B**) stained with methylene blue. Ultramicrograph of the esophagus (**C–H**) A: pseudostratified epithelium (ep). Mesenchymal condensation in the lamina propria (lp). (**B**) Myoblasts (m) aggregation at the prospective site of the muscular layer. Note telocytes (arrowheads). (**C**) Pseudostratified epithelium had superficial mucous-secreting cells (blue colored). Putative dendritic cell (golden color) had vesicles (v) and granules (gr). Interstitial cell (brown color). (**D**) Superficial mucous-secreting cells (blue colored) contained mucous granules (gr). (**E**) Epithelial cell contained RER (r), ribosomes (ri), mitochondria (m). (**F**) Putative dendritic cell (golden color) had vesicles (v) and granules (gr). (**G**) Interstitial cell (brown) had dilated RER (r), note telopodes of telocyte (blue). (**H**) Myoblasts (pink) aggregation surrounded by telocytes (blue). Note telopodes (arrowheads).

**Figure 9:**
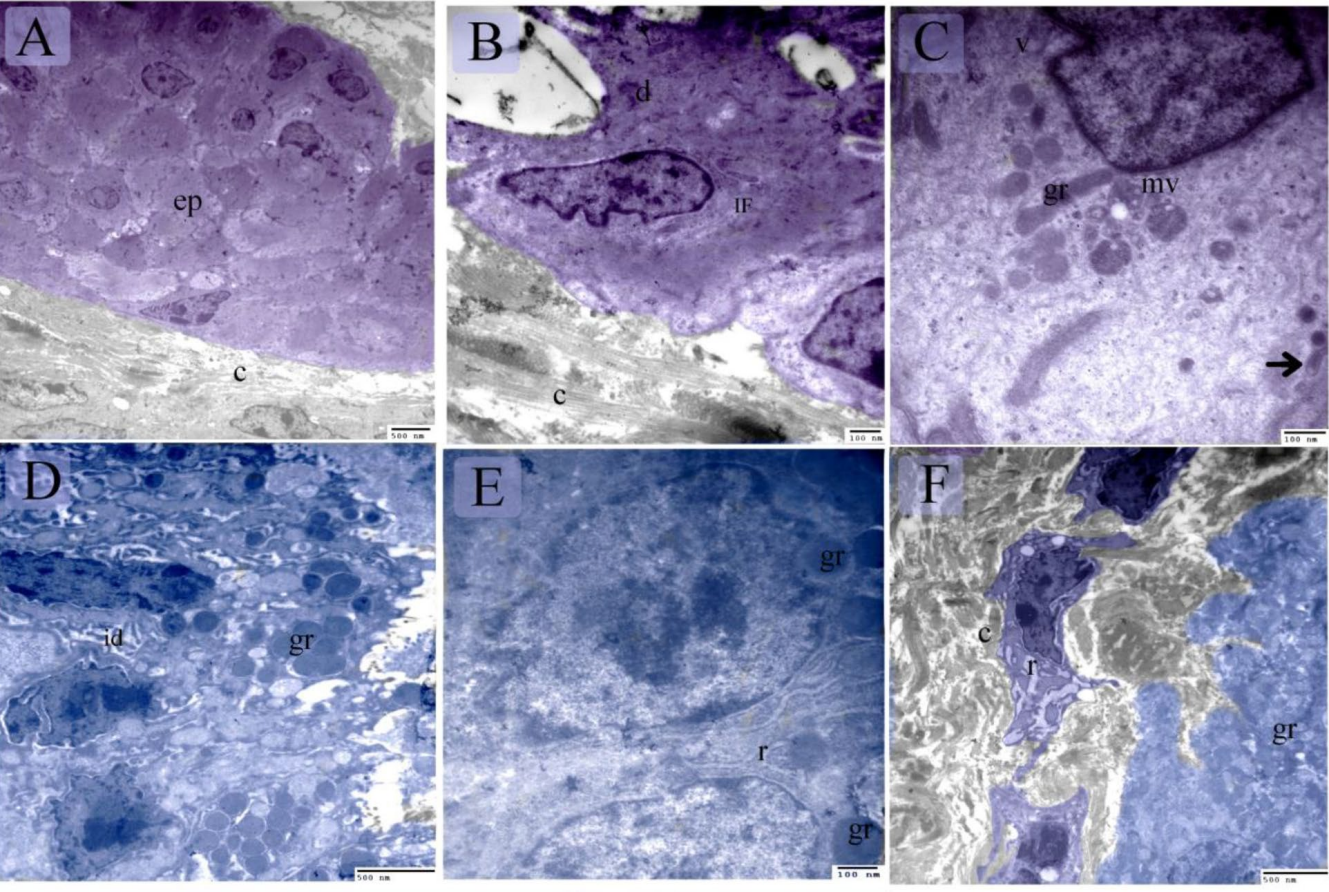
Ultrastructure features at day 17. (**A**) A part of an invaginated epithelium (ep). Note collagen fibers (c). (**B**) Keratinocyte had less keratin intermediate filament (IF), desmosomes (d), collagen fibers (c). (**C**) Dendritic cells had vesicles (V), multivesicular bodies (mv), dense granules (gr), rod-shaped granule (arrow). (**D**, **E**) Esophageal gland contained mucous granules (gr), RER (r), interdigitation (id). (**F**) Fibroblast (dark blue) rich in dilated RER (r). Collagen (c), granules of the mucous gland (gr).

The pseudostratified epithelium and the basal lamina appreaed PAS-positive on the 8th day of incubation (Fig. 6A–C). The developing muscular layers were distinguished by the myoblasts (Fig. 6D), some of which contained PAS-positive glycogen granules. PAS-positive myelin sheaths were also detected between myoblasts (Fig. 6D). On the 15th day of incubation, the thoracic portion of the esophagus was lined by pseudostratified epithelium exhibited PAS activity while the gland was inactive PAS-ve (Fig. 6E–G,J). PAS-ve collagen fibers were identified in the submucosa and muscular layer (Fig. 6G,K). Telocytes exhibited high affinity for PAS (Fig. 6G). The esophageal mucous glands in the cervical portion were now active and contained PAS-positive granules (Fig. 6H,I,L,M). On the 13th day of incubation, epithelial invaginations established sac-like glandular units. The epithelium was pseudostratified type (Fig. 7A–C) and had mucous-secreting inclusions that appreaed metachromatic by toluidine blue but the gland was inactive exhibited -ve PAS reaction (Fig. 7A–E). Mitotic figures were detected in the interstitial cells (Fig. 7B), epithelial cells (Fig. 7C), and SMC (Fig. 7F). On the 15^th^ day of incubation, the esophagus exhibited distinct mucosal foldings, and the epithelium was now stratified squamous non-keratinized. (Fig. 7G,H). The esophageal mucous glands in the cervical portion were now active (Fig. 7G,H). TEM imaging revealed that the pseudostratified epithelium had superficial mucous-secreting cells (Fig. 8A–D) and the myoblasts developed (Fig. 8B). Most epithelial cells contained RER, ribosomes, and mitochondria (Fig. 8E), and putative dendritic cells had vesicles and lamellar granules (Fig. 8F). The lamina propria contained interstitial cells rich in dilated RER (Fig. 8G). The developing muscular layers were distinguished by the myoblasts (Fig. 8H). On the 17th day of incubation, the esophageal keratinocytes developed keratin intermediate filaments (Fig. 9A,B). Dendritic cells appeared between keratinocytes. Dendritic cells were recognized by vesicles, multivesicular bodies, dense granules, and rod-shaped granules (Fig. 9C). Esophageal glands contained mucous granules, RER, and exhibited interdigitation (Fig. 9D,E). Collagen fibers were identified using TEM (Fig. 9A,B,F).

**Figure 10:**
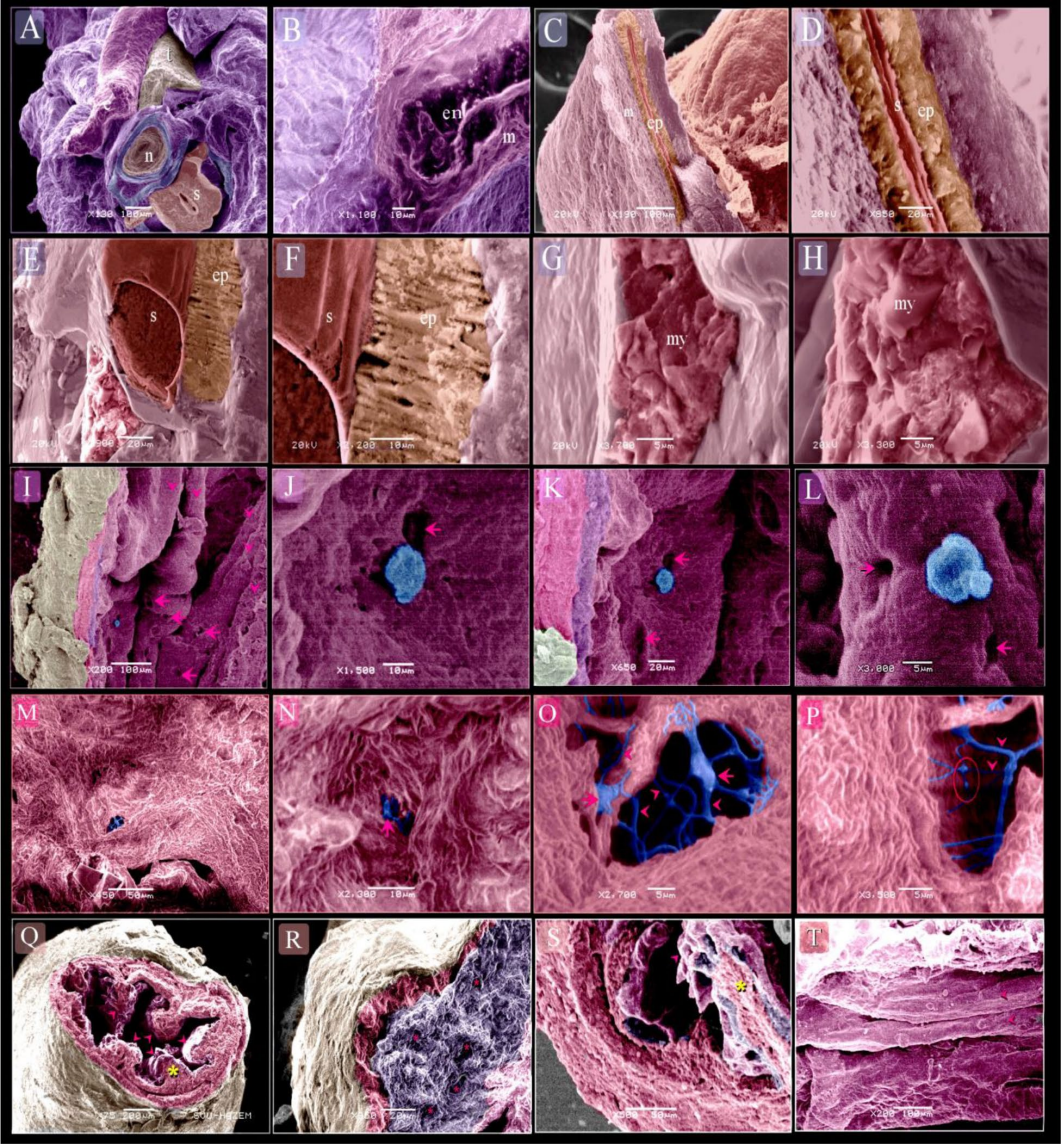
Scanning electron microscopic features of the embryonic esophagi at day 5 (**A**, **B**), at day 8 (**C**–**H**), 14 (**I**–**L**) and 17 (**M**–**T**). (**A**) Cross section of the neck showed esophagus (e), trachea (t), spinal cord (s), notochord (n). (**B**) Endoderm (en) and mesoderm (m). (**C**, **D**) Cross section of the esophagus. pseudostratified epithelium (ep). Secretion (s). Mesenchyme (m). (**E**–**H**) Longitudinal section of the esophagus. pseudostratified epithelium (ep). Secretion (s). myoblasts (my). (**I**–**L**) SEM of day 14. Surface epithelium (purple), Lamina propria (violet), Muscle (Pink), Serosa (yellow), multiple opening (arrows) of the esophageal glands. Mucous secretion (blue). Mucosal folds (arrowheads). (**M**) Colored scanned image of day 17 showed telocytes between the muscle bundles. (**N–P**) Higher magnification of image E. note the cell body of Telocyte (arrow), thick and thin telopodes (arrowheads), podomes (red circle). (**Q**, **S**) Cross section of the esophagus showed mucosal folds (arrowheads). Epithelium (violet), muscularis mucosae (yellow asterisk), muscular layer (pink), serosa (pale brown). (**R**, **T**) Lamina propria (blue) contained esophageal glands (pink asterisks), muscular layer (pink), serosa (pale brown). (**F**) Scanned mucosal surface showed mucosal folds (arrowheads).

On the 5th day of incubation, scanned samples of the esophagus showed the presence of endoderm and mesoderm and the endoderm was pseudostratified epithelium (Fig. 10A,B). On the 8th day of incubation, the pseudostratified epithelium was also existed (Fig. 10C–F). Development of the myoblast layer occurred at this age (Fig. 10E–H). On the 14th day of incubation, the esophagus formed a distinct mucosal fold which contained the opening of the esophageal glands (Fig. 10I–L), and on the 17th day of incubation (Fig. 10Q,S,T) the esophageal glands were now visible in the lamina propria (Fig. 10R). TCs were identified in the muscular layer (Fig. 10 M–P).

**Figure 11:**
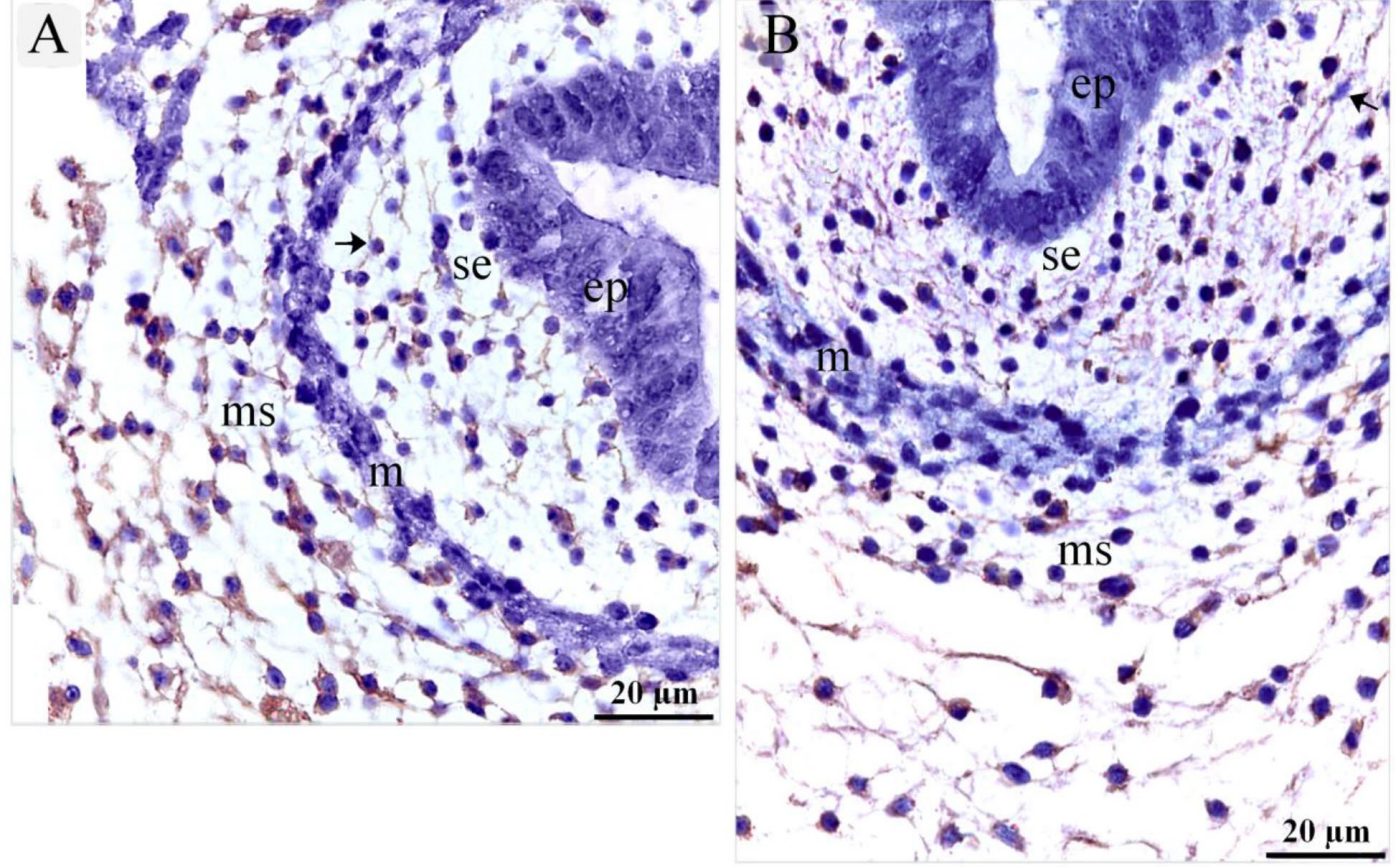
Immunohistochemical staining of the esophagus at day 5 using CD34 and VEGF. Immunostained paraffin sections of day 5 for CD34 (**A**) and VEGF (**B**). (**A**) CD34 positive cells were identified in subepithelial tissue (se) and mesenchyme (ms). Strong CD34 positive TCs (arrows) were distinguished by cell prolongations (telopodes). Note the epithelium (ep), myoblasts (m). (**B**) VEGF positive cells were identified in the subepithelial tissue (se), and mesenchyme (ms). VEGF positive TCs (arrows) were distinguished by cell prolongations (telopodes). (**C**) CD34 positive cells were identified in the epithelium (ep), lamina propria (LP). Note the epithelium (ep), myoblasts (m).

**Figure 12:**
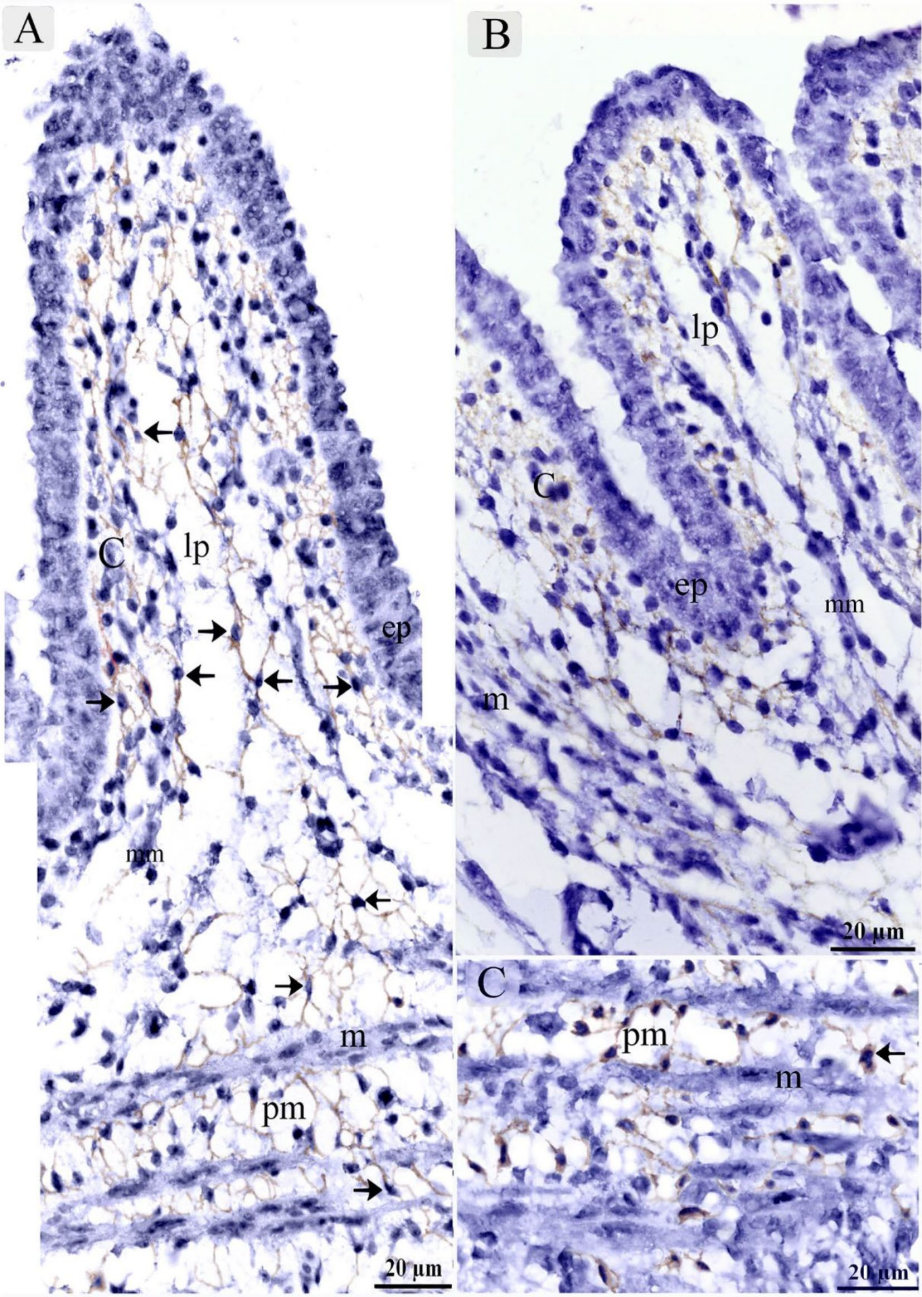
Immunohistochemical staining of the esophagus at day 8 using CD34 and VEGF. Immunostained paraffin sections of day 8 for CD34 (**A**) and VEGF (**B**). (**A**) Strong CD34 positive cells were identified in the condensed layer (c) of the lamina propria (lp) and peri-muscular tissue (pm). Strong CD34 positive TCs (arrows) were distinguished by cell prolongations (telopodes). Note muscle cells (m). (**B**) VEGF positive cells were identified in the lamina propria (lp) and peri-muscular tissue (pm). VEGF positive TCs (arrows) were distinguished by cell prolongations (telopodes). Note muscle cells (m).

**Figure 13:**
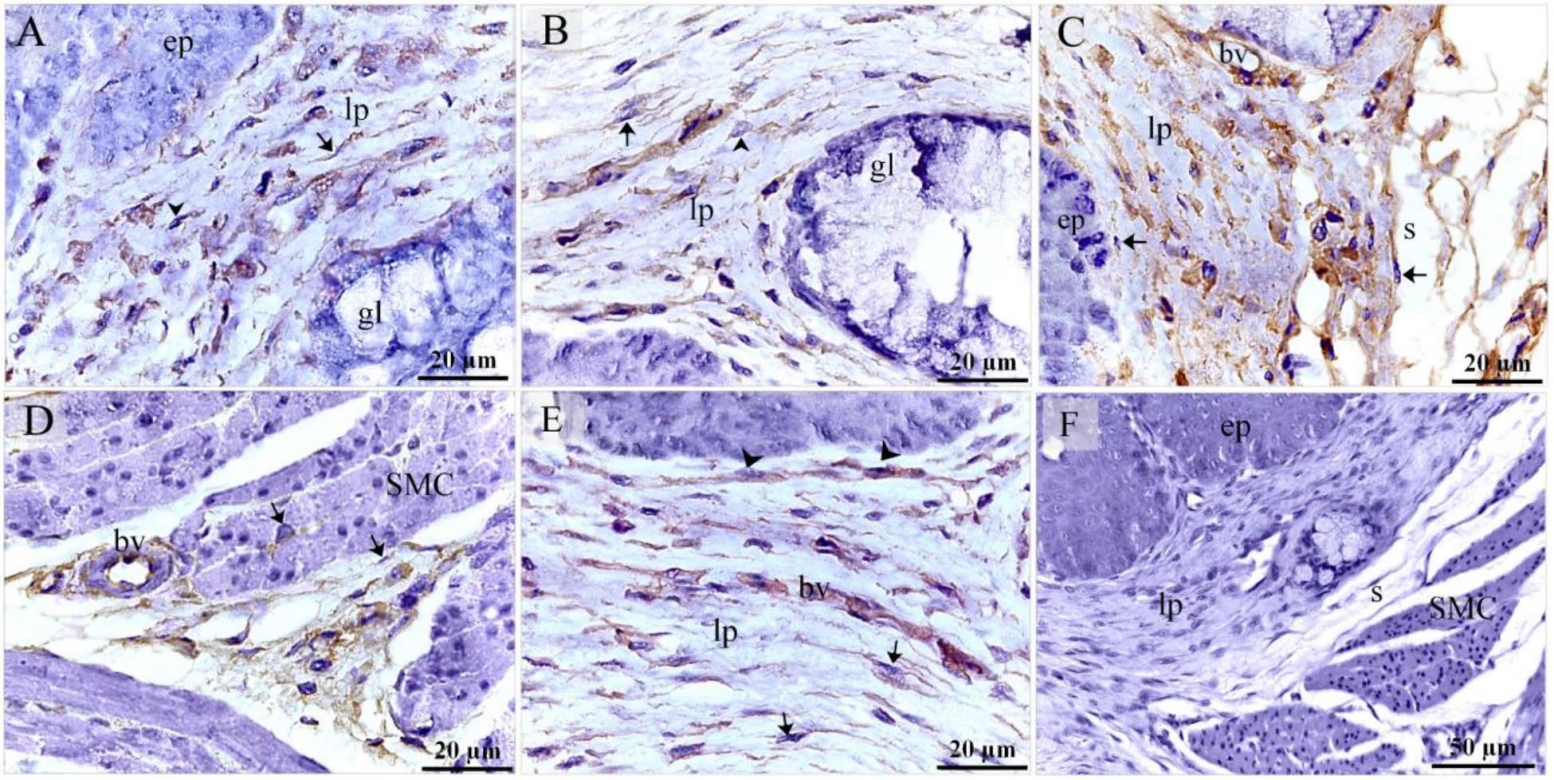
Immunohistochemical staining of the esophagus at day 15 using CD34. Immunostained paraffin sections for CD34. (**A**, **B**) Interstitial cells in the lamina propria (lp) were CD34-positive. CD34-positive TCs (arrows) were distinguished by cell prolongations (telopodes). CD34-positive fibroblast-like cells were detected. Note the epithelium (ep) and the gland (gl). (**C**) Numerous interstitial cells in the lamina propria (lp) were CD34-positive. Note the epithelium (ep), blood vessel (bv) and submucosa (s). CD34-positive TCs (arrows) were distinguished by cell prolongations (telopodes). (**D**) Peri-muscular tissue (pm) had strong affinity for CD34. CD34-positive TCs (arrows) were distinguished by cell prolongations (telopodes). Endothelial cells lined the blood vessel (bv) were CD34-positive. (**E**) Sprouting endothelial cells (arrowheads) located in lamina propria (LP). Note blood vessel (bv), CD34 positive TCs (arrows). (**F**) Negative control for CD34 immunostaining. Note epithelium (ep), lamina propria (lp), serosa (s), muscular layer (m).

On the 5th day of incubation, CD34 and VEGF positive cells were identified in the subepithelial tissue and the mesenchyme. CD34 positive TCs were distinguished by cell prolongations or telopodes and VEGF positive TCs were recognized as well (Fig. 11A,B). On the 8th day of incubation, it is notable that CD34 positive cells were more obvious in the lamina propria compared to VEGF. CD34 and VEGF positive cells were also identified in the peri-muscular tissue. CD 34 positive and VEGF positive TCs were distinguished by their telopodes (Fig. 12A–C). The sprouting endothelial cells were CD34 positive (Fig. 13E). Figure 13 F represented negative control for CD34.

**Figure 14:**
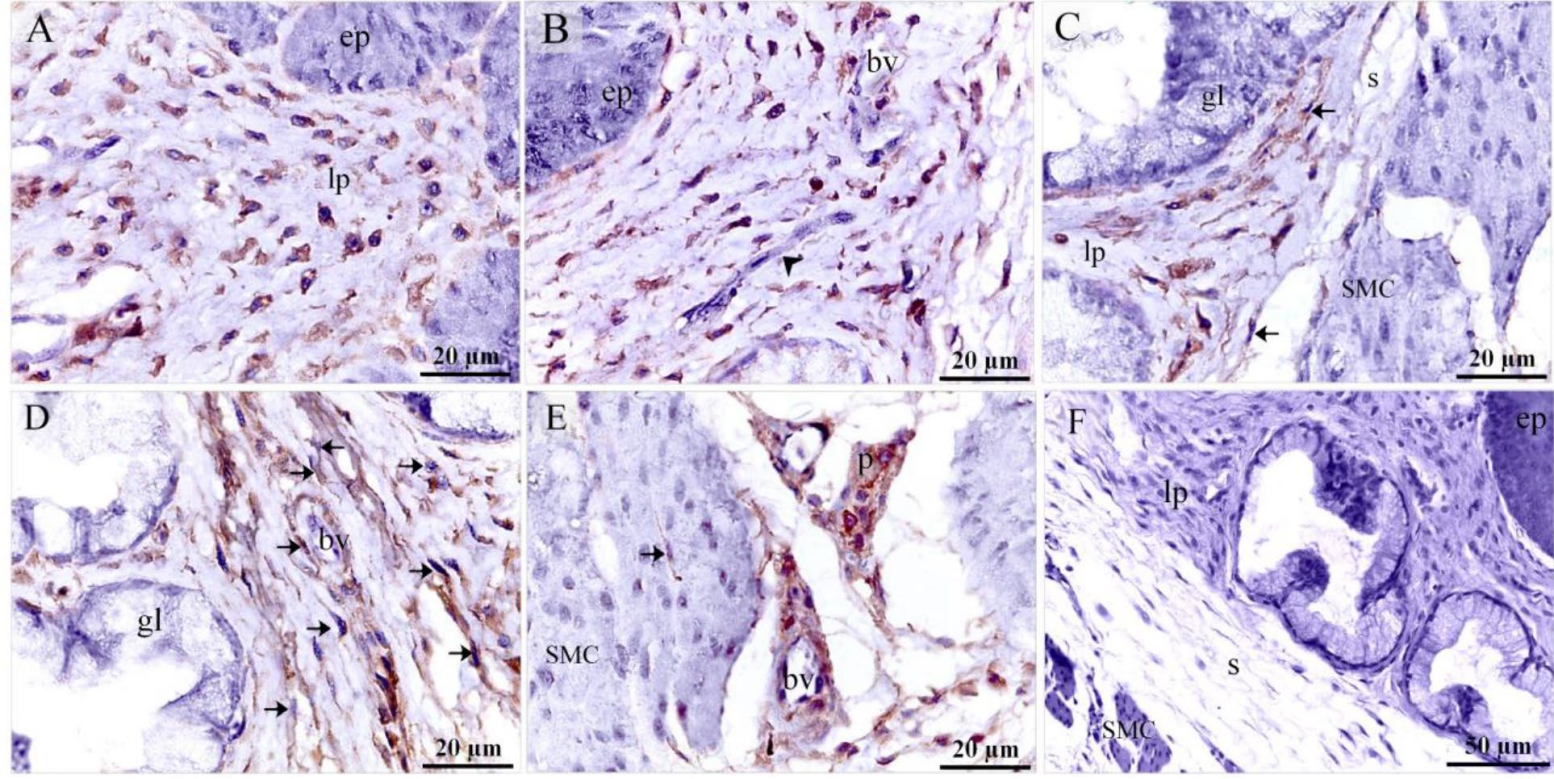
Immunohistochemical staining of the esophagus at day 15 using VEGF.Immunostained paraffin sections for VEGF. (**A**) Interstitial cells in the lamina propria (lp) were VEGF positive. Note the epithelium (ep). (**B**) Sprouting endothelial cells (arrowhead) were VEGF-postive. (**C**) Interstitial cells in the lamina propria (lp) were VEGF positive. VEGF-positive TC (arrow) was distinguished by their telopodes. Note gland (gl), submucosa (s), muscle (m). (**D**) Lamina propria (lp) rich in TCs (arrows). Note gland (gl), blood vessel (bv). (**E**) Perivascular tissue (p) was VEGF-positive. Note blood vessel (bv), muscle (m). VEGF-positive TC (arrow) was distinguished by their telopodes. (**F**) Negative control for VEGF immunostaining. Note epithelium (ep), lamina propria (lp), serosa (s), muscular layer (m).

On the 15th day of incubation, Interstitial cells in the lamina propria and the submucosa were CD34 positive including TCs that had distinct telopodes, and fibroblasts which had voluminous cytoplasm and short cell processes (Fig. 13A–D). Perivascular tissue between muscles had strong CD34 immunoaffinity (Fig. 13D). CD34-positive TCs were detected between muscle fibers and bundles (Fig. 13D). Interstitial fibroblast-like cells in the lamina propria were VEGF positive (Fig. 14A). Endothelial sprouting in the lamina propria were VEGF positive (Fig. 14B). VEGF-positive TCs were identified in the lamina propria and the submucosa (Fig. 14C,D). Perivascular tissue had strong immunoaffinity for VEGF (Fig. 14E). Figure 14 F represented negative control for VEGF.

**Figure 15:**
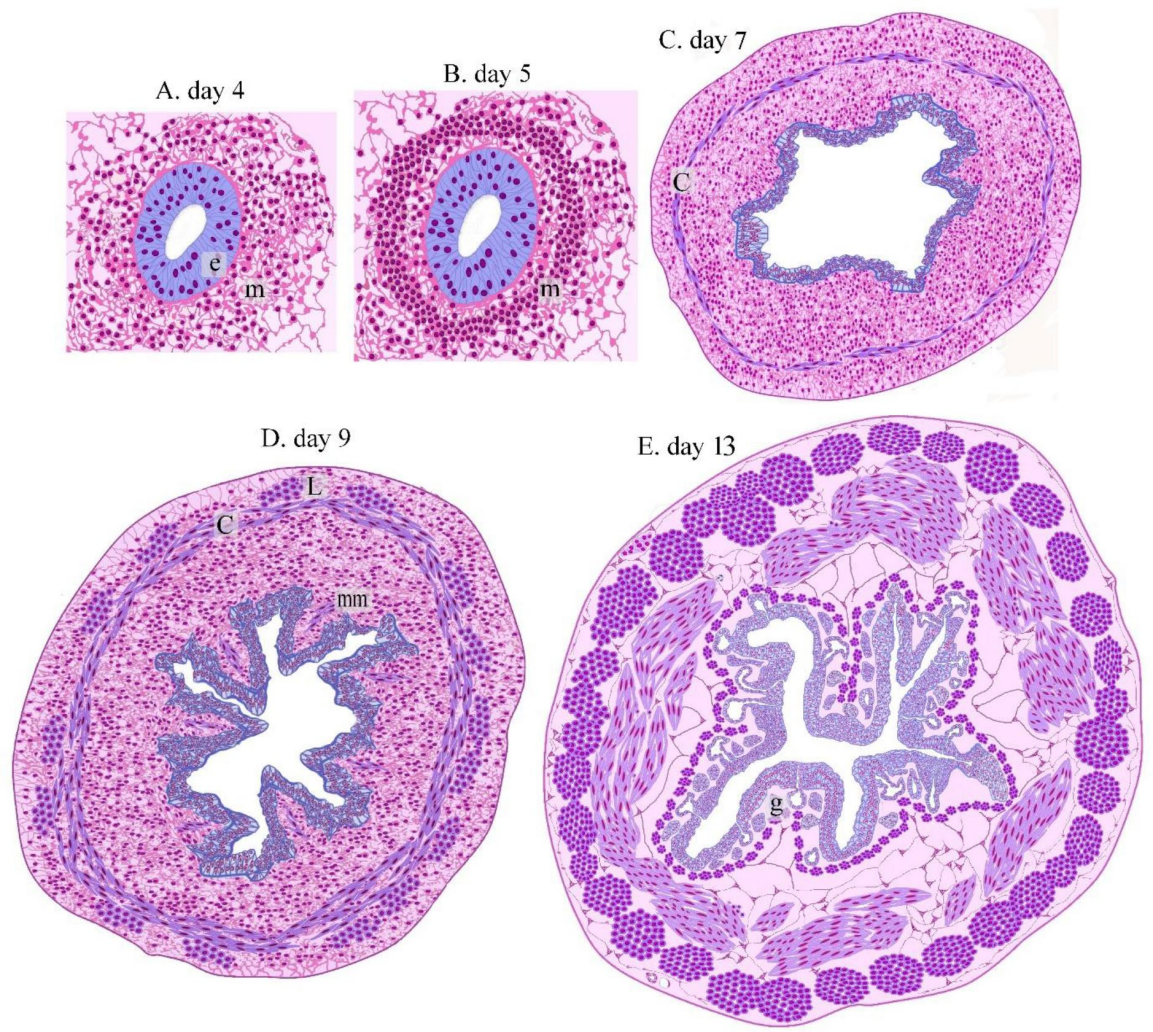
Illustration of sequences of histological events occurring during esophageal development. (**A**) Day 4. The gut tube was formed, consisting of endoderm (e) and mesoderm (m). (**B**) Day 5. mesenchymal cell condensation (m) occurred as a sign for development of the muscular layer. (**C**) Day 7. Development of the inner circular SMC (c). (**D**) Day 9. development of the inner circular SMC (c). outer longitudinal (L) and muscularis mucosae (mm). (**E**) Day 13. Development of the gland (g).

All events that occurred during esophageal development were summarized in (Fig. 15).

## Discussion

The current study investigated histological events occurring during the development of the esophagus of the quail.

The gut tube was formed on the 4th day of incubation. This was composed of endoderm and covered by mesoderm, which was previously described by another study^52^. The authors mentioned the vacuolated stage, in which extracellular vacuoles appeared between the epithelial cells, and they suggested that the vacuoles may be related to the developmental cysts.

Mesenchymal condensation was the first sign of development of the muscular layer. Development of the muscular layer occurred in a sequential manner; the inner circular layer on the 7th day, the outer longitudinal layer on the 8th day, and the muscularis mucosae began by mesenchymal condensation and development of strands of SMC on the 9th day. A similar sequence of muscular development occurs in the pheasant esophagus; development of the inner circular layer of the begins on the 8th day, the outer longitudinal layer on the 12th day, and the muscularis mucosae is completed on the 18th day^53^. The sequence of muscular coat development of the human embryos is also similar to avian species. However, in the Guinea Fowl (Numida meleagris). The tunics of the muscularis are not well developed till at posthatch^54^. The muscular layers of the human esophagus develop in similar sequence to quail. In the 12 mm and 20 mm crown-rump stages human embryos, the inner muscular layer become more developed. The outer longitudinal muscle layer and the muscularis mucosae are prominent^55^.

In the current study, PAS-positive inclusions were detected in some myoblasts and myelin sheaths. PAS is used to identify the simple sugars in myelin^56^. The study used Bielschowsky's silver stain to visualize the SMC, which exhibited a granular appearance indicating dense bodies. Visualization of the myofilaments striations in skeletal muscles was documented by using Bielschowsky’s stain^57^.

Esophageal epithelium directly transformed from pseudostratified columnar into the stratified squamous non-keratinized type by the 15th day. In all stages of esophageal development, the epithelium was non-ciliated that may be a unique feature of quail esophagus. A different types of epithelium is noted in the pheasant; pseudostratified columnar epithelium is observed on the 9th day of incubation and transformed into stratified cuboidal by the 10th day, then to simple cuboidal epithelium between the 10th and 12th days, and finally to stratified squamous on the 13th day^53^. In the chukar partridge, the epithelium transformed to 2 layered cuboidal cells with some cilia by day 9 of incubation. The ciliated cells increased during embryonic development on day 10, 11. By day 18 of incubation, two types of simple and pseudostratified columnar epithelial tissue are detectable in the thoracic portion of esophagus. The cilia were difficult to distinguished due to the epithelial secretion^58^. Developmental variations of the esophagus are documented in human embryos. The esophageal epithelium is stratified columnar of four cells deep in the 8.5 mm crown-rump human embryo and become multilayered columnar in the 40 mm crown-rump stage. Epithelium is surrounded by undifferentiated mesenchyme and condensed cells form the inner muscle coat in the 8.5 mm crown-rump embryo. In the 30 to 40 mm crownrump embryo, the basal epithelial cells protrude toward the lumen to become ciliated columnar cells. The ciliated cells develop in the middle third of the esophagus and extend towards the cranial and caudal portions. The ciliated cells distribute along the entire mucosa of the esophagus of the 60 mm crown-rump embryo, except for the upper and lower ends. The ciliated cells gradually disappear with proceeding the development and only isolated patches of ciliated columnar cells occasionally remain until after birth^55^. Development of ciliated esophageal epithelium is associated with transformation into stratified columnar in type. Formation of ciliated stratified columnar epithelium occurs on the 8th week of gestation and were decreased after the14th week. The stratified columnar epithelium transforms into the stratified squamous epithelium during the 4th month^59^.

Glandular development began on the 13th day of incubation. Epithelium invaginated into the underlying connective tissue, forming sac-like glandular units. A similar occurrence can be observed in chickens, starting on the 12th day of incubation^60^. In pheasants, glandular development begins as epithelial buds in the esophagus on the 18th day of incubation^53^. The current study revealed epithelial transformation preceded glandular activation. The undifferentiated pseudostratified epithelial cells the differentiated into keratinocytes and dendritic cells. Esophageal glands which synthesize polysaccharide components were detected using PAS and toluidine blue. Through the metachromatic reaction from toluidine blue, some of these components were identified as glycosaminoglycans (GAGs), which the avian esophageal glands secrete along with mucopolysaccharides^53,60^. The submucosa of the quail esophagus was a delicate layer. Similar to other avian species, very thin layer of submucosa is described in the pariah kite, median egret, goshawk, dove and duck^61^, the House Sparrow^62^, and chukar partridge (Alectoris chukar)^58^. In pigeons, the submucosa is thin in female (40.2±7.5 in the cervical region and 20.6± 3.6 in the thoracic region) and is relatively thicker in male (60.7±11.5 in the cervical region and 40.6±8.9 in the thoracic region)^63^. While, the submucosa in the esophagus of human^64^ and small animals^65^ is a thick layer.

In the current study, we focused on the morphological novelties of quail esophagus and discussed the possible role in adaptation to their environments. Embryonic esophageal epithelium of quail didn’t acquire cilia. Unlike, other avian and mammalian species^55,58,59^. The development of the cilia is related to the functional requirements. It is not clear whether the cilia are motile true cilia that remove the surface secretions/foreign particles or non-motile stereocilia that has absorptive function. In chick embryos, the esophageal cilia associate with microvilli and microplicae^66^. Esophageal cilia have been described as transitory structures in avian and mammals in contrast to the lower vertebrates such as the crocodiles. Esophagus of the juvenile American alligators (*Alligator mississippiensis*) has ciliated columnar epithelium^67^. It is interesting that embryonic quail esophagus lacked the skeletal muscles, unlike the higher (mammalian) and lower species (aquatic and reptiles). The esophagus is devoid of striated muscles in wild avian species such as Rock Dove, Collared Dove, Rose-ringed Parakeet, Kestrel, House Sparrow and Linnet^68^*,* mallard, spot-billed duck, Ural owl and Hodgson's hawk-eagle^69^, and other birds, Kingfisher (*Halcyon smyrnensis*) and Hoopoe (*Upupa epops*)^70^ and chicken^71^. Thus, it seems that involuntary control of the esophagus is unique feature of avian species. This may be correlated to feeding habitat that requires peristatic involuntary movement during regurgitative feeding to feed their young. It seems that regurgitation may require definite time lapsing to deliver *a* semi*-*digested food. In mammals, the muscular wall of the esophagus is comprised of both skeletal and smooth muscles. However the distribution of the two types varies according to animal species^72,73^. Among fish, the esophagus of *Anablepsoidesurophthalmus* has some striated muscle fibers^74^. While, heavy striated musculature thick inner longitudinal and outer circular layer are described in tilapia^75^, Trachelyopterus striatulus (Siluriformes: Auchenipteridae)^76^. Krauss et al. explain the development of the esophageal muscular layer with the variable degree of esophageal striated myogenesis. Esophageal striated muscle progenitor cells that originate in the craniopharyngeal mesoderm and express the early marker *Mesp1*, and subsequently express *Tbx1* and *Isl1*. Esophageal striated muscle progenitor cells colonize the proximal portion of the esophageal muscular layer. They migrate distally along the esophageal muscular layer forming the transition zone where they express Pax7, Myf5 and MyoD, and myogenin. Some Pax7^+^ and Myf5^+^/MyoD^+^ cells propagate to generate adequate numbers of precursor cells for the muscular layer. Some differentiate into striated myofibers, which form proximal to the transtion zone. The authors descried a linear expression pattering Isl1, Pax7, Myf5/MyoD progression^77^.

The architecture of the esophageal glands of avian and mammals are quite similar. Mammalian esophageal glands are compound tubuloalveolar type in mammals^73^ and some birds such as barn owl (*Tyto alba) and* common wood pigeon^78^, and compound tubular type in wild bronze turkey (Meleagris gallopavo)^79^. The avian esophageal glands modify that have short ducts, and located in the lamina propria. While, mammalian esophageal glands are more developed dominated the submucosa^69^. The ducting system have some of characteristics different according animals species^73^. It is noted that esophageal mucous glands progressively develop with higher classes of the evolution tree. The geometric parameters of esophageal submucosal glands are measured in the avian and porcine species. The esophageal submucosal glands comprised 35% in avian and 45% in porcine area of the submucosa. The glands have an area of 125,000 μm^2^ in avian) and 580,000 μm^2^ in porcine^80^. Some fish esophagi lack the esophageal glands, instead mucous cells are located in the epithelium. This occurs in *Sphoeroides testudineus*^81^, *Trichomycterus brasiliensis*^82^ and *Anablepsoidesurophthalmus*^74^, Nile tilapia and African catfish^75^, Larimichthys crocea (Acanthopterygii: Perciformes)^83^. While, other aquatic fish, the Asian seabass (*Lates calcarifer*), has both goblet cells and esophageal glands^84^. In crocodiles, the juvenile American alligators (*Alligator mississippiensis*), the esophageal epithelium has few mucous goblet cells^67^. The mucous secretion is essential to moisten food, lubrication and have protective function to neutralize the gastric acidity in case gastric reflux^85^. The evaluation of the stomach and the specification of gastric epithelium is described among fishes, amphibians, reptiles, birds and mammals^86^. Comparisons of stomach acidity in mammal and bird indicates that scavengers and carnivores exhibit higher stomach acidities compared to herbivores or carnivores feeding on phylogenetically distant prey^87^. Delicate submucosa is a characteristic feature of avian esophagus. This may be related to absence of the submucosal glands. unlike mammals, the abundant glandular subunits occupied the submucosa in dogs while extends to the tunica muscularis in rabbit^73^.

The current study detected localization of collagen and reticular and elastic fibers in embryonic quail esophagus. Staining with Mallory trichrome and Weigert-Van Gieson revealed the presence of collagen fibers in the basal lamina, lamina propria, between the muscle fibers, and the serosa. Reticular fibers were detected in the lamina propria, myocytes, around glands, and in the myenteric or Auerbach's plexus. The observation of reticular fibers around the SMC was supported by previous studies^88^. Van-Geison stain was used to detect collagen in the pheasant^53^ and geese^89^ esophagus, particularly in the lamina propria and submucosa of the latter. The amount of collagen fibers in the avian esophagus depended on the feeding habits of the species. The lamina propria had more collagen fibers in pariah kites, egrets, and doves, while only a few collagen and reticular fibers were detected in goshawks and ducks. Collagen and elastic fibers are found in the tunica adventitia of the esophagus of pariah kites, median egrets, goshawks, doves, and ducks^61^.

Putative dendritic cells were found in the pseudostratified epithelium. Vesicles and granules were observed on the 8th day of incubation. Active dendritic cells were identified after epithelial stratification, and they contained vesicles, multivesicular bodies, dense granules, and rod-shaped granules. Dendritic cells act as antigen-presenting cells and contribute to the adaptive and innate immune response^76–92^. Dendritic cells were recognized using TEM by the presence of the dendritic lamellar and multivesicular bodies^93,94^. They also express MHC class II antigen which stimulates naïve T-lymphocytes and initiates the primary immune response. They are also involved in the activation of B-cells and reactivation of the memory B lymphocytes^90,92^.

In the current study, we used CD34, a common marker for undifferentiated stem cells, to demonstrate the distribution of different types of putative stem cells during the different stages of esophageal development. On the 5th day of incubation, CD34 positive cells were identified in the, subepithelial tissue, and mesenchyme, while on the 8th day they were also identified in the lamina propria and peri-muscular tissue. On the 15th day. The, interstitial cells including TCs and fibroblasts, exhibited CD34 immunoreactivity. Telocytes have an essential role in morphogenesis, embryogenesis, organization of the embryonic tissues^81–97^, cell migration, cell adhesion, proliferation, differentiation^33,35,96^, tissue homoeostasis, remodeling^98^ and repair^99^. The CD34 protein is a member of a family of transmembrane sialomucin proteins. CD34 is common for marker of hematopoietic stem cells, hematopoietic progenitor cells, as well as non-hematopoietic cells such as vascular endothelial progenitors and embryonic fibroblasts, multipotent mesenchymal stromal cells, interstitial dendritic cells, and epithelial progenitors. CD34 is critical for adhesion molecules^100^, proliferation and blocking differentiation of stem or progenitor cells^87–103^.

Angiogenesis is the physiological process of forming new blood vessels from the pre-existing vessels. Growth of the vasculature is associated with proliferation of the endothelial cells to form sprouting vascular branches. Angiogenic events are essential during development to provide adequate nutrients for the microenvironment required by cells and tissues. If the oxygen levels are low, cells may experience hypoxia. Hypoxic cells express vascular proliferation markers to induce and enhance angiogenesis^104,105^. In the current study, we used immunohistochemistry to demonstrate the types of cells that express VEGF, a vascular proliferation marker. On the 5th day of incubation, VEGF positive cells were identified in the subepithelial tissue, and mesenchyme, and on the 8th day of incubation, they were also identified in the lamina propria and peri-muscular tissue. VEGF positive TCs were distinguished by their telopodes. On the 15th day of incubation, Telocytes may promote endothelial cell proliferation and angiogenesis^106^. Stromal fibroblasts express VEGF^107^. VEGF is secreted by the salivary gland and also shed in the saliva. Salivary VEGF is an essential stimulus for oral mucosal tissue repair^108,109^.

## Conclusion

The current study provided information on esophageal development, including a timeline of the development events, and described changes of cellular components. The quail esophagus may be used as a model to study esophageal disorders. The current study also discussed the unique morphological features of quail esophagus and analyze the evolutionary morphology among different classes in relation to the function.

## Abbreviations

SMC: Smooth muscle cells
NBF: Neutral buffered formalin
PAS: Periodic acid-schiff stain
TC: Telocyte
VEGF: Vascular endothelial growth factor
